# Monocytes and macrophages and placental malaria infections in an area of unstable malaria transmission in eastern Sudan

**DOI:** 10.1186/1746-1596-6-83

**Published:** 2011-09-19

**Authors:** Magdi M Salih, Amal H Mohammed, Ahmed A Mohmmed, Gamal K Adam, Mustafa I Elbashir, Ishag Adam

**Affiliations:** 1 Faculty of Medical laboratory Sciences, University of Khartoum, Khartoum, Sudan; 2 Faculty of Medicine, Ribat University, Khartoum, Sudan; 3 Faculty of Medicine, Gadarif University, Gadarif, Sudan; 4 Faculty of Medicine, University of Khartoum, Khartoum, Sudan

## Abstract

**Background:**

Maternal immunity is thought to play a major role in the increased susceptibility of pregnant women to *Plasmodium falciparum *malaria. Few studies exist on immunohistochemical characterization of the placental inflammatory infiltrate. The current study was conducted in Gadarif hospital in an area characterized by unstable malaria transmission in eastern Sudan.

**Method:**

Ninety three placentae were investigated for malaria histological changes and immunohistochemical study for monocytes and macrophages (CD68).

**Results:**

While 1(1.1%), 2(2.2%) and 20(21.5%) of the 93 placentae had acute, chronic and past malaria infections, 70(75.2%) had no malaria infections. Monocytes and macrophage (CD 68) were detected in 29 (31.2%) of these 93 placentae. Significantly higher rate of monocytes and macrophage were detected in placentae with malaria infections [11/23 (47.8%) vs. 18/70 (25.7%); *P *= 0.047] especially in placentae with past malaria infections. Placental malaria infections and monocytes and macrophages cells infiltration were not different between primiparae and multiparae. There was no significant difference in the birth weight between the women with placental malaria infections/monocytes and macrophages cells infiltration and those who had no placental malaria infections/cellular infiltrations.

**Conclusion:**

Significantly higher rate of monocytes and macrophage were detected in placentae with malaria infections. Neither placental malaria infections nor cellular infiltrates were associated with parity or lead to reduction of birth weight.

## Introduction

Malaria during pregnancy is a major public health problem in tropical and subtropical regions; each year 25 million African women become pregnant in malaria endemic areas [1]. Pregnant women are more susceptible to malaria than their non-pregnant counterparts [2]. Malaria infections are associated with poor maternal and fetal outcomes [3,4]. Malaria during pregnancy is a huge burden in Sudan [3,5] and it is one of the leading causes of maternal mortality [6].

During pregnancy, adhesion of *Plasmodium falciparum*-infected erythrocytes to syncytiotrophoblast leads to parasite sequestration in the intervillous space. The parasite adheres specifically to chondroitin sulfate-A expressed on syncytiotrophoblast [7]. The increased susceptibility of pregnant women to malaria was thought to result from pregnancy-related immunomodulation and Th1/Th2 shift to decreased Th1-type cytokines and increased Th2-type cytokines to prevent rejection of fetal allograft [8,9]. However, this modulation was proposed to result from a state of monocyte activation and lymphocyte inhibition [10], the immunomodulation is more important in placental than in the peripheral blood [11]. The inflammatory response is responsible for functional damage in placental villi, and disturbs feto-maternal exchange, leading to low birth weight [12,13]. Histological studies on malaria have shown that *P. falciparum*-infected placentae are characterized by an increase in inflammatory cells in the intervillous space [13,14]. The placental malaria parasite -related cell infiltrates are mainly monocytes and macrophages, with a smaller population of granulocytes and lymphocytes [14-17]. Few studies addressed the characteristics of the immunological responses of these cell infiltrates [18]. In the present study, we identified monocytes and macrophages in the placenta immunohistochemically, using monoclonal antibodies to CD68, in samples from women in Gadarif hospital which is located in an area characterized by unstable malaria transmission in eastern Sudan [19].

## Materials and methods

A cross sectional study was conducted in Gadarif Maternity Hospital during October 2009. Ninety-three consecutive women with singleton pregnancy were approached to participate in the study. After signing an informed consent, obstetrical and medical history (age and parity) were gathered using questionnaires. The babies were weighed immediately following the delivery using electronic digital scale to the nearest 50g. Maternal, placental and cord blood films were prepared, the slides were Giemsa-stained and the number of asexual *P. falciparum *parasites per 200 white blood cells was counted and double-checked blindly by an expert microscopist. Maternal haemoglobin concentrations were estimated by HemoCue haemoglobinometer (HemoCue AB, Angelhom, Sweden).

### Placental Histology

**Figure 1:**
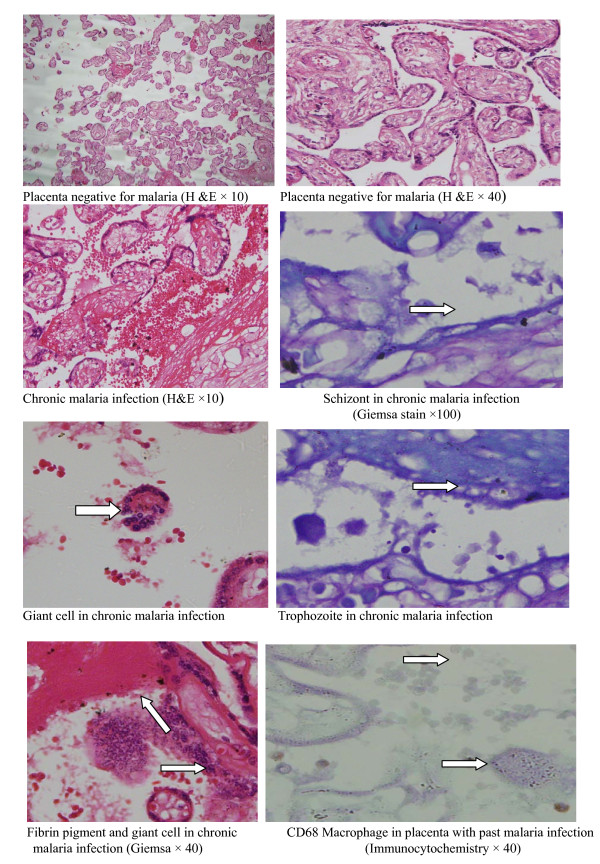
**Placental histology, malaria infections and monocytes/macrophages infiltrates**.

The details of this have been shown before [5,20]. In summary in all women, approximately a three cm^3 ^sample was removed from the maternal surface in an off-center position, half the distance between the umbilical cord and the edge of the placenta. Once collected, each biopsy sample was placed in 25 mL of 10% neutral buffered formalin. All biopsy samples were kept at room temperature and were stored in Gadarif until transportation to Khartoum, where the histologic studies were performed. The placental biopsy samples were then processed and were embedded in paraffin wax, by standard techniques. In every case, paraffin sections 4 mm thick were stained with hematoxylin-eosin and Giemsa stain. Because the samples were fixed in buffered formalin, formalin pigment formation, which has similar optical characteristics and polarized light activity to malaria pigment was not detected [21]: Placental malaria infections were characterized based on the classification of Bulmer *et al *[16]: uninfected (no parasites or pigment), acute (parasites in intervillous spaces), chronic (parasites in maternal erythrocytes and pigment in fibrin or cells within fibrin and/or chorionic villous syncytiotrophoblast or stroma), past (no parasites and pigment confined to fibrin or cells within fibrin), Figure 1.

### Immunohistochemical methods

Immunohistochemical analysis was performed in neutral formalin-fixed, paraffin-embedded tissue, using the IHC-Tek™ Avidin/Biotin Blocking Solution following the manufacturer's instructions http://www.ihcworld.com/products/IHC-Tek-Reagent.htm. In summary, 4-mm sections were deparaffinized and were hydrated through xylene and graded alcohols, and peroxidase was blocked for 5 min in 0.03% H_2_O_2 _containing sodium azide. Then the slides were incubated with the primary antibody for 40 min against CD68. The peroxidase-labeled polymer was then applied for 40 min. After washing in TBS, the slides were incubated with the diaminobenzidine substrate chromogen solution, washed in distilled water, counterstained with hematoxylin, washed, dehydrated, and mounted. A pressure cooker was used for heat-induced epitope retrieval with antibodies [22]. Monocytes and macrophage inflammatory cells quantification was performed with an Olympus microscope at magnification of 40 × using an eyepiece with a field of view of 26.5 mm. The numbers of monocytes and macrophages CD68 cells results were expressed as geometric mean and standard deviation cell count per square millimeter.

### Statistics

Data were entered in computer using SPSS for windows version 16.0 for analysis. Student's t-test and X^2 ^were used to compare means and proportions between the groups, respectively.

### Ethics

The study received ethical clearance from the Research Board at the Faculty of Medicine, University of Khartoum.

## Results

### General characteristics

The age of these 93 women ranged from 13-44 with the mean (SD) of 25.9 (7.3) years. Of these 93 women, 34 (36.5%) were primiparae. Only 12 (13.0%) gave history of using bed nets and none of these 93 women used malaria prophylactic treatment during the index pregnancy. There was one blood film positive in maternal and placental set. Of these 93 women, 47 (50.5%) were anaemic (haemoglobin < 11 g/dl) and 7 (7.5%) had low birth weight deliveries.

### Placental histology and malaria infections

Placental histology showed that 1 (1.1%), 2 (2.2%) and 20 (21.5%) had acute, chronic and past malaria infections, respectively and 70 (75.2%) had no malaria infections. Of 34 primiparae, 8 (23.5%) vs. 15 (25.4%) of the 59 multiparae had placental malaria infection, *P *= 0.838. The rate of anaemia was not different between women with placental infections and those women who had no placental infections, 12/23 (52.2%) vs. 35/70 (47.3), *P *= 0.944. There was no significant difference in mean (SD) birth weight between the women with placental malaria infections and those who had no placental malaria infections, 2962.5(455.5) vs. 3035.7 (538.4) gm; *P *= 0.576.

### Immunohistochemical analyses

The monocytes and macrophage cell infiltrations were detected in 29 (31.2%) of the placentae. The number of monocytes and macrophages ranged from one to fifteen cell with the geometric mean (SD) of 3.1(3.3) cells/mm^2^. Of 34 primiparae, 11(32.3%) vs. 18 (30.5%) of the 59 multiparae, *P *= 0.853 had placental monocytes and macrophage cell infiltrations.

Significantly higher rate of monocytes and macrophages infiltrates were detected in placentae with malaria infections, 11/23 (47.8%) vs. 18/70 (25.7%); *P *= 0.047, table 1. The majority of these monocytes and macrophages were detected in placentae with past malaria infections (10/20, 50%) than in placentae with acute (0/1, 0%) and chronic infections (1/2; 50%). The mean (SD) birth weight (3016.9 (441.3) vs. 3018.5(551.2) gm; *P *= 990) and the rate of low birth weight deliveries was not different between those who had placentae with monocytes and macrophages infiltrates and those who had placentae without cellular infiltrate, table 1.

**Table 1:** Histology, immunohistology and pregnancy outcomes of 93 placentae in eastern Sudan

Variables	Positive for cellular infiltrations (N = 29)	Negative for cellular infiltrations (N = 64)	*P*
Primiparae	11(38.0)	23(36.0)	0.853
Histology			
Uninfected	18(62.1)	52(81.2)	0.029
All infections	11(38.0)	12(18.8)	0.047
Acute infection	0(0)	1(1.6)	0.579
Chronic infection	1(3.4)	1(1.6)	0.579
Past infection	10(34.5)	10(15.6)	0.040
Anaemia	20(69.0)	27(42.2)	0.005
low birth weight	2(6.8)	5(7.8)	0.876

## Discussion

This is the first study to investigate malaria placental histology and monocytes and macrophage cellular infiltrations in an area with unstable malaria transmission in Africa. The main findings of the current study were; most (20/23, 87.0%) of the placental infections were past infections which affect pregnant women regardless to their parity and had no effects on birth weight. Monocytes and macrophages cellular infiltrations were detected in 32.2% of the placentae. They were more predominant among placentae with past malaria infections irrespective to parity, associated with maternal anaemia and had no effect on birth weight. We have previously shown that placental malaria infections, hormonal and cytokines levels were not different between the primigravidae and multigravidae among pregnant women in eastern and central Sudan [20,23,24]. This observation could be explained by the low immunity among pregnant women in an area of unstable malaria transmission.

In neighboring Tanzania, it has been shown that, malaria parasitized placentae, especially in primigravidae, had the most significant increase in all inflammatory cellular types -except NK cells-with monocytes and macrophages representing the major population of the infiltrate [18]. It has been previously shown that the inflammatory response was particularly marked in chronic placental malaria infections, no increase in inflammatory cell counts were observed in cases with past infection and these infiltrates were associated with reductions in birth weight [18]. Likewise, Ismail et al., [15] observed that primiparae had higher placental infections, chronic infections and inflammatory cell infiltration more frequently than multiparae. In their observation; chronic malaria infection had the significant inflammatory cell infiltration, acute infections showed a mild increase in inflammatory cell infiltration and those with past infections had no increase in the cell infiltration. However, the low prevalence of placental malaria in these women in the current study, the relatively small sample size and perhaps the size of placental tissue itself makes it hard to compare this study to other ones of placental malaria. Because malaria (past or present) was not very common, and because chronic infections were very uncommon (and these chronic infections are the ones associated with heavy monocyte infiltrates and poor outcomes in previous studies), the power of this study to examine malaria associated changes is rather limited. Furthermore we introduce the presence/absence of CD68 cells as another way of stratifying the data; it is not clear what finding these cells in low numbers means (the normal number of these cells is not known), especially in the absence of malaria. There is a certain percentage of CD68+ cells in the blood in normal subjects, so there will be some chance of finding one or more of these cells on a normal placental section. Many other studies have reported increased inflammatory cells infiltration mainly monocytes and macrophages in placental malaria infections [17]. These inflammatory cells might have an important role in *P. falciparum *clearance and phagocytosis of the infected red blood cells. On the other hand these inflammatory cells might lead to functional damage in placental villi, and disturb feto-maternal exchange, leading to low birth weight [12,13]. The mean birth weight was not different between women with placental malaria infection/with monocytes macrophages infiltrates and those women without placental malaria infections/cellular infiltrates. This goes with the previous observations where the placental malaria infections were not associated with low birth weight in eastern Sudan [5,20]. The lack of association between malaria infections and low birth weight might be explained by the small sample size of these studies and the lack of power. However, these cellular infiltrates were associated with reduction in birth weight [13] and malaria infections were known to be associated with low birth weight [12]. Due to fund constraints, only the CD 68 marker for monocytes and macrophages was investigated in the current study. The other marker e.g. CD20 and other inflammatory cells (B, T lymphocytes were not investigated. However, Ordi et al. [13] reported that, malaria parasitized placentae, especially in primigravidae, had the most significant increase in all inflammatory cellular types (except NK cells) and these infiltrates were associated with reduction in birth weight.

## Conclusion

Significantly higher rate of monocytes and macrophage were detected in placentae with malaria infections. Neither placental malaria infections nor cellular infiltrates were associated with parity or lead to reduction of birth weight.

## Competing interests

The authors declare that they have no competing interests.

## Authors' contributions

MMS and IA designed the study, GKA and MIE conducted the clinical work. MMS, AHM, MIE and AAM conducted the lab work. IA and GKA participated in the statistical analyses. All the authors approved the draft and the final paper.
